# Sertraline-Induced Galactorrhoea: A Case Report

**DOI:** 10.7759/cureus.62357

**Published:** 2024-06-14

**Authors:** Jihane Moussaoui, Mohammed Barrimi

**Affiliations:** 1 Psychiatry, Faculty of Medicine and Pharmacy Oujda, Mohammed First University, Oujda, MAR; 2 Immunohematology and Cellular Therapy Laboratory, Faculty of Medicine and Pharmacy, Mohammed First University, Oujda, MAR

**Keywords:** prolactin, depression, sertraline, galactorrhoea, antidepressant

## Abstract

Patients taking various treatments frequently report galactorrhoea as a side effect. Psychotropic drugs, especially neuroleptics, are among the treatments most likely to cause this effect. Conventional tricyclic antidepressants rarely cause galactorrhea. The advent of new selective serotonin reuptake inhibitor (SSRI) antidepressants purported to reduce such side effects. We report the clinical case of a patient with galactorrhoea on Sertraline as well as our therapeutic approach in light of data from recent scientific literature.

## Introduction

Selective serotonin reuptake inhibitors (SSRIs) are preferred not only for their efficacy but also because they are considered more tolerable and safe than other antidepressants used for anxiety and depression [1]. Frequent use of these drugs can lead to various side effects.

Galactorrhoea is defined as a lactescent discharge through the nipple in the absence of pregnancy and at a distance from breastfeeding (>6-12 months). It is not the result of physiologic lactation; it is the result of hyperprolactinemia caused by medications or many medical conditions [1]. Hyperprolactinemia can be found in pituitary tumours, hypothalamic lesions, chronic renal failure, polycystic ovary syndrome, cirrhosis, and hypothyroidism, and sometimes the cause is not identified [1,2].

Galactorrhoea is an adverse effect frequently reported with psychotropic drugs, especially neuroleptics. Publications reporting galactorrhoea on antidepressants are limited, especially selective serotonin reuptake inhibitors such as sertraline. This latter is considered one of the best-tolerated antidepressants. It can be found in both female and male patients [3].

We report the case of a patient with galactorrhoea on sertraline by comparing it with some cases reported in the scientific literature to highlight the need for close clinical monitoring for the detection of adverse reactions when initiating antidepressant treatment.

## Case presentation

We report a case of a 21-year-old female patient who is married and working as a nurse. She has no pathological history or personal or family psychiatric history. The patient is not medicated and has never taken antidepressants before.

The spontaneous expression of a death wish prompted her to consult the psychiatric emergency unit with her father. She was hospitalized in the psychiatric department for passive suicidal ideation. Her symptomatology dates back to a month before the gradual installation of isolation, anorexia, insomnia, sadness of mood, loss of pleasure, disinvestment of her usual activities, and remarks of low self-esteem with verbalization of a desire for death.

At the psychiatric examination, the patient is conscious, well-oriented in time and space, hypomimic, and calm on the motor level, and her higher functions seem to be preserved. She presented with bradyphaemia, with easy contact, a sad mood, a concordant, non-anxious affect, verbalizing a loss of pleasure and interest, words of devaluation, despair, and underestimation, without any delirious themes or perceptual disorders, and passive suicidal ideation, insomnia, and anorexia evoking a major depressive episode.

The patient's physical examination was normal. Her blood pressure was 130/80 mmHg, her heart rate was 79 beats per minute, and her respiratory rate was 20 cycles per minute. Her body mass index was 24 kg/m^2^. The mammary gland examination was normal. Her electrocardiogram was normal. The patient was initially given sertraline 50 mg/day with a progressively decreasing anxiolytic (alprazolam 0.5 mg/day during seven days, then 0.25 mg/day during seven days, then we stopped the treatment.) combined with psychotherapy sessions.

On the third day of treatment, the patient presented bilateral galactorrhea compared to the pre-therapeutic clinical examination with no other associated clinical signs. She did not present amenorrhoea or signs of intracranial hypertension, so her complete biological assessment with beta human chorionic gonadotropin (HCG) is unremarkable, as presented in Table 1. We have also included the results of an assessment done six months previously during a routine medical examination.

**Table 1:** The results of the patient's biological assessments FSH: follicle-stimulating hormone, LH: luteinizing hormone, TSH: thyroid-stimulating hormone, ASAT: aspartate aminotransferase, ALAT: alanine aminotransferase, GGT: gamma-glutamyl transferase, PAL: alkaline phosphatase, LDH: lactate dehydrogenase, HCG: human chorionic gonadotropin

Parameters	Patient's current values	Patient's values ​​from 6 months ago	Normal values
Prolactin	6.5 ng/ml	6.2 ng/ml	3–25 ng/ml
FSH	5.1 mIU (follicular phase)	5.5 mIU (follicular phase)	Follicular phase: 3.3–10 mIU/ml ovulatory phase: 6–20 mIU/ml Luteal phase: 2–10 mIU/ml menopause: 41–124 mIU/ml
LH	3.6 mIU (follicular phase)	4.1 mIU (follicular phase)	Follicular phase: 2–12 mIU/ml ovulatory phase: 19–90 mIU/ml Luteal phase: 0.7–12 mIU/ml menopause: 20–92 mIU/ml
Urea	0.22 g/l	0.19 g/l	0.15–0.39 g/l
Creatinine	7.3 mg/l	7.1 mg/l	5.7–11.1 mg/l
TSH	1.89 μIU/ml	1.86 μIU/ml	0.35–4.94 μIU/ml
ALAT	32 IU/l	36 IU/l	0–55 IU/l
ASAT	20 IU/l	25 IU/l	5–34 IU/l
GGT	21 IU/l	19 IU/l	9–36 IU/l
PAL	96 IU/l	106 IU/l	40–150 IU/l
LDH	133 IU/l	146 IU/l	125–220 IU/l
White blood cells	7600 /μl	8200/μl	4000–10,000/μl
Hemoglobin	14 g/dl	14 g/dl	12–16 gd/l
Platelet count	250,000/μl	220,000/μl	150,000–400,000/μl
Glycemia	0.71 g/l	0.80 g/l	0.70–1.10 g/l
Beta HCG	0.60 IU/l	0.72 IU/l	<5 IU/l

Brain magnetic resonance imaging (MRI) returned no abnormality, as did an abdominopelvic ultrasound. We stopped treatment with sertraline and continued Alprazolam. We noticed a disappearance of the galactorrhea after four days of stopping.

We discussed the diagnosis of induced galactorrhea by referring to several arguments: absence of pathological history, normal clinical examination, normal brain and abdominal imaging and biological assessment, absence of amenorrhoea, the appearance of galactorrhea after the introduction of treatment and its improvement upon cessation of treatment, and the absence of an aetiology of galactorrhea before the introduction of treatment.

Our patient was put on venlafaxine 75 mg/day, and we increased the dose five days later to 150 mg/day with good tolerance and a satisfactory clinical evolution in relation to her depression up to 12 months of regular follow-up in psychiatric consultation.

## Discussion

To discuss the diagnosis of galactorrhea induced by sertraline, it is necessary to eliminate all other possible and sometimes rare causes of this condition. The clinical examination of our patient and the normal biological and radiological assessment allowed us to eliminate the usual causes of galactorrhea. These pathologies can either induce this symptom or predispose to its occurrence if a medication is added.

The particularity of our clinical case lies not only in the nature of the incriminated molecule but also in its period of appearance. There are certainly some similar cases reported in the literature. Doruk and Dogrul reported a similar case of a female patient with galactorrhea on sertraline, which improved on discontinuation and switching to venlafaxine. The chronological evolution of this effect suggests an iatrogenic origin linked to sertraline in this case, as in ours [2].

Compared to sertraline, which is little incriminated, other SSRIs have also been implicated in the occurrence of galactorrhea, in particular scitalopram, escitalopram, paroxetine, and fluoxetine [2].

To explain this undesirable effect, it is necessary to understand that galactorrhea seems to be mediated by the serotonergic activation of prolactin-releasing factors, such as the thyrotropin-releasing hormone of the postsynaptic 5HT receptors of the hypothalamus, and/or by the serotonergic inhibition of prolactin-releasing hormone (PRL)-releasing factors such as dopamine, or indirectly via the release of oxytocin or vasoactive intestinal peptide [3]. The iatrogenic galactorrhea with normal prolactin levels appears to be due to indirect inhibition of tuberoinfundibular dopaminergic neurons [4]. Also, genetic mechanisms such as serotonin or dopamine receptor polymorphisms may play a role in the emergence of SSRI-induced extrapyramidal side-effects (EPS) [5].

This adverse effect remains little mentioned, and no clinical trial about galactorrhea has been carried out or published; however, a few rare cases have been reported, including one associating galactorrhea with oro-mandibular dystonia reported by Doruk and Dogrul [2,5-7].

It is important to ensure that galactorrhea is due to medication and not to a structural lesion in the hypothalamic or pituitary area or other medical conditions. This can be accomplished by stopping the medication temporarily, reducing the dose to determine whether it returns to normal, or, in some cases, switching to a medication that does not cause galactorrhea and performing biological tests, magnetic resonance imaging, or computed tomography of the hypothalamic and pituitary areas to eliminate other causes [8,9]. The management of galactorrhea depends on its aetiology.

## Conclusions

Conducting antidepressant treatment requires close monitoring of tolerance and efficacy in order to detect any adverse effects. This assessment must be done on the first day of treatment. Galactorrhea induced by antidepressants, particularly sertraline, may resolve spontaneously upon stopping treatment. Its persistence should lead to the search for other aetiologies, probably aggravated by sertraline. The adverse effects should always be explained to patients so that they can detect them and consult their doctor as soon as possible.

## References

[1] Bruehlman RD, Winters S, McKittrick C (2022). Galactorrhea: rapid evidence review. Am Fam Physician.

[2] Doruk N, Dogrul BN (2021). Sertraline induced oromandibular dystonia and galactorrhea: a case report. Psychiatr Danub.

[3] Verhelst J, Abs R (2003). Hyperprolactinemia: pathophysiology and management. Treat Endocrinol.

[4] Nicholas L, Dawkins K, Golden RN (1998). Psychoneuroendocrinology of depression. Psychiatr Clin North Am.

[5] Özten E, Hizli Sayar G, Göğçegöz Gül I, Ceylan ME (2015). Sertraline Induced Galactorrhea. Noro Psikiyatr Ars.

[6] Emiliano AB, Fudge JL (2004). From galactorrhea to osteopenia: rethinking serotonin-prolactin interactions. Neuropsychopharmacology.

[7] Mahasuar R, Majhi P, Ravan JR (2010). Euprolactinemic galactorrhea associated with use of imipramine and escitalopram in a postmenopausal woman. Gen Hosp Psychiatry.

[8] Hedenmalm K, Güzey C, Dahl ML, Yue QY, Spigset O (2006). Risk factors for extrapyramidal symptoms during treatment with selective serotonin reuptake inhibitors, including cytochrome P-450 enzyme, and serotonin and dopamine transporter and receptor polymorphisms. J Clin Psychopharmacol.

[9] Molitch ME (2005). Medication-induced hyperprolactinemia. Mayo Clin Proc.

